# 

**DOI:** 10.1017/S0950268819000918

**Published:** 2019-05-29

**Authors:** Lucy J. Robertson

**Affiliations:** Department of Food Safety and Infection Biology, Faculty of Veterinary Medicine, Norwegian University of Life Sciences, Oslo, Norway

At some point in their studies, nearly all medical students are informed that the Rod of Asclepius, the symbol of a snake wound around a staff used to signify medical practice, and that forms the centre piece of the World Health Organization (WHO) logo, actually represents a Guinea worm, *Dracunculus medinensis*, on a removal stick. There seems to be no evidence for this proposal, and, indeed, there are plenty of other more plausible suggestions. However, it is perhaps good to keep the Guinea worm theory alive, as otherwise medical students of today may not hear of this parasite, and dracunculiasis, the disease it causes, at all.

As of 2017, WHO had certified 1186 countries as free of dracunculiasis, leaving only four countries endemic, two countries at the precertification stage and two not known to have the disease but yet to be certified. In 2018, just 27 cases were reported. But it has not always been so, with around 3.5 million cases registered in the mid-1980s. In 2013, five countries, all in Africa, gained the status of being free of dracunculiasis – one of these was Nigeria.

The route for a country to move from being endemic to certified free of infection is not necessarily a smooth or easy one. In this book, Professor Luke Ekundayo Edungbola, who has been intimately connected with the eradication of this parasitic infection in Nigeria, provides some personal insights into the ups and downs of such an endeavour.

The book is written in a very personal style, and is full of adventure, excitement, and opinions – and one can only be impressed by Professor Edungbola's dedication to achieving the eradication goal. At different points, he describes the campaign against Guinea worm as a fierce battle, and there is certainly something of the flamboyant warrior in the descriptions of his sometimes alarming adventures – several of which concerned vehicle mishaps, but also encounters with all kinds of personalities, including criminals, as he and his team travel around Nigeria following up cases or outbreaks, distributing necessary supplies and collecting data. Often, it would seem, they use their own resources and money to keep the fight going. The detailed descriptions of day-to-day activities indicate that Professor Edungbola must have made copious notes on what each skirmish involved.

Beginning with an overview of the fundamentals of Guinea worm, and dracunculiasis, with descriptions of his own first encounters, this is an engaging and easy read. And there are many illustrative photographs, not only of cases of dracunculiasis with worms protruding from limbs, but also of other people and places encountered and relevant in the campaign, maps and many other things beside. Although a light editorial hand from Elsevier could have been judiciously applied – the name of the parasite is misspelled in the very first paragraph of Chapter 1, and Figures 1.1 and 2.2 are identical – much of the pleasure from reading this book is the energy and personality shining through every description and each event. Although not all readers may agree that a deity has been involved in the work described, Professor Edungbola nevertheless seems sure that a higher power has been instrumental in Nigeria achieving the WHO certification of being free of dracunculiasis.

Thus, this is not a text book in any formal sense of the word, but an old-fashioned adventure story of the very best kind, in that, despite the many obstacles, the hero comes out victorious in the end.

